# Aggressive Endovascular Management of Massive Dural Venous Sinus Thrombosis in the Setting of Acute Myeloid Leukemia

**DOI:** 10.7759/cureus.3891

**Published:** 2019-01-15

**Authors:** Ali S Haider, Hasan Sumdani, Justin McCaslin, Ahmed Habib, Kennith F Layton

**Affiliations:** 1 Neurosurgery, Texas A&M College of Medicine, Houston, USA; 2 Neurosurgery, Texas A&M College of Medicine, Round Rock, USA; 3 Radiology, Baylor University Medical Center, Dallas, USA; 4 Neurosurgery, The University of Texas MD Anderson Cancer Center, Houston, USA

**Keywords:** neurosurgery, interventional neuroradiology, thrombectomy, dst, acute myeloid leukemia, endovascular, aml, dural sinus thrombosis, coagulopathy

## Abstract

While hematologic malignancies are understood to be a risk factor for dural sinus thrombosis (DST), little data is available regarding the presentation and management of synchronous cases. In this case, a 40-year-old woman with newly diagnosed acute myeloid leukemia (AML) developed extensive DST with symptoms refractory to systemic anticoagulation. The decision was made to pursue aggressive endovascular intervention and the patient’s symptoms significantly improved with minimal residual deficits. Here, we report the clinical course and complex management of this rare clinical scenario.

## Introduction

Dural sinus thrombosis (DST) is a rare disease process which can be difficult to diagnose, and once discovered it can be even more challenging to manage. A variety of risk factors for DST have been described, including hematologic malignancies [1-3]. We present the case of a 40-year-old woman with newly diagnosed acute myeloid leukemia (AML) who while admitted to the hospital for induction chemotherapy was subsequently found to have developed extensive DST. The complex medical and interventional management is discussed while the literature on this topic is also reviewed.

## Case presentation

**Figure 1:**
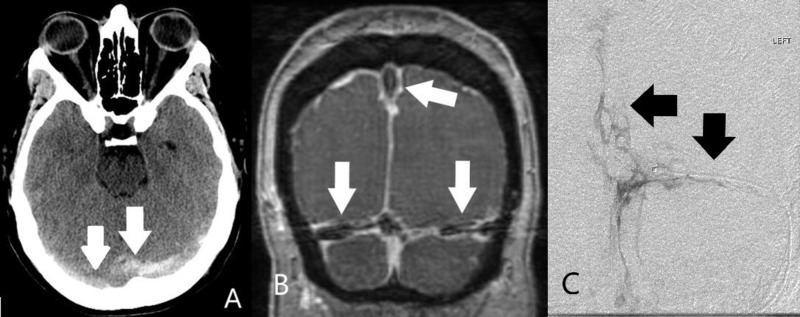
Diagnostic confirmation of multiple dural sinus thromboses with several imaging modalities. A) Noncontrast head computed tomography scan demonstrating hyperdense appearance of the bilateral transverse sinuses (arrows). B) Coronal post-contrast magnetic resonance venography at the torcula demonstrating extensive filling defects throughout the dural sinuses (arrows). C) Digital subtraction angiography image from catheter venography demonstrating extensive filling defects through the dural sinuses (arrows).

A 40-year-old woman with newly diagnosed AML undergoing induction chemotherapy developed blurry vision on admission day 16. Findings of bilateral papilledema and severe retroorbital headaches on day 18 prompted a computed tomography (CT) scan of the head which was unrevealing. Persistent symptoms prompted a repeat CT on day 27 which revealed findings concerning for DST (Figure 1A).

This was further shown by magnetic resonance venography (MRV) to involve the bilateral transverse sinuses (TSs), superior sagittal sinus (SSS) and straight sinus (Figure 1B). Systemic anticoagulation via continuous IV heparin drip was immediately initiated. Despite medical therapy, the patient's symptoms continued to worsen.

**Figure 2:**
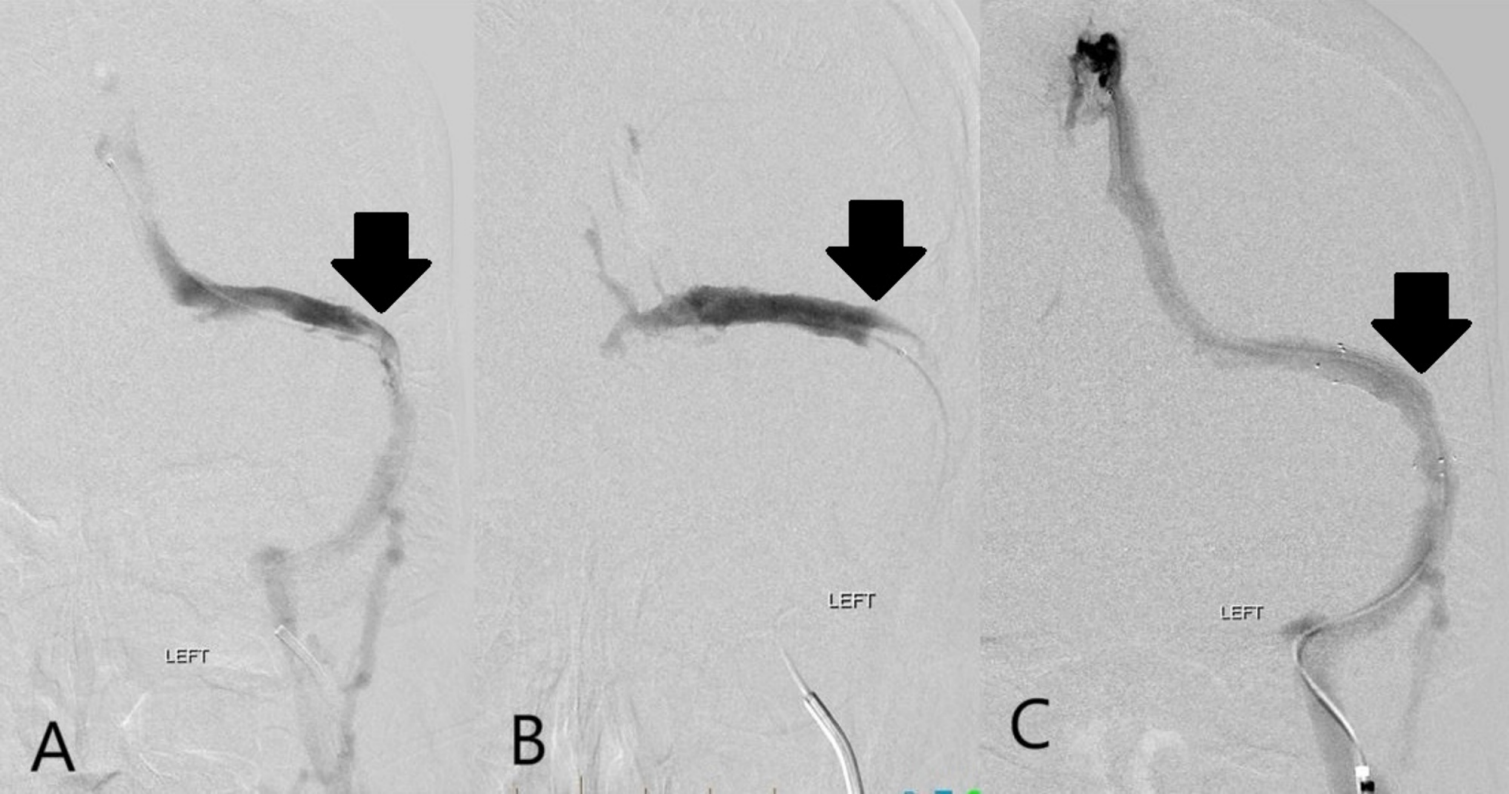
Rethrombosis of transverse sinus and subsequent treatment with angioplasty and stent placement. A) Morning of day 30 digital subtraction angiography image from left transverse sinus venography demonstrating partial recanalization of the left transverse sinus with limited outflow (arrow). B) Evening of day 30 digital subtraction angiography image demonstrating rethrombosis of the left transverse sinus with complete occlusion (arrow). C) Evening of day 30 digital subtraction angiography image demonstrating restoration of outflow after angioplasty and left transverse sinus stent placement (arrow).

The patient was taken to the angiography suite on day 28, where extensive DST was confirmed by catheter venography (Figure 1C). Following venography, an alteplase drip was placed via infusion microcatheter (Renegade^TM^ Hi-Flo^TM^, Boston Scientific, USA) in the dominant left TS and infused overnight. The following day, the patient returned to the angiography suite where repeat catheter venography revealed multiple, small, and irregular channels within the thrombus of the left TS. However, a very large clot burden was still noted, which precluded access of the SSS. The alteplase infusion microcatheter was again advanced into the large burden of clot within the left TS and infusion resumed throughout the day. Later the same day, repeat catheter venography revealed a recanalized left TS but with persistent outflow obstruction. The microcatheter was then successfully advanced into the SSS where it was left for overnight alteplase infusion at the same rate (Figure 2A).

On the morning of admission day 30, the patient again returned to the angiography suite where venography revealed persistent large clot burden within the same distribution and rethrombosis of the left TS. Mechanical thrombectomy was then performed using a 6 mm x 30 mm stent retriever (Solitaire^TM^, Medtronic, USA). A large volume of adherent clot was successfully removed from the left TS, however, a large clot burden remained. Final venography during this intervention revealed a partially recanalized left TS and partial underlying outflow obstruction thought due to either focal stenosis or a venous web at the junction with the left sigmoid sinus. Large volume clot persisted within the SSS, therefore the alteplase infusion microcatheter was left in the SSS for infusion throughout the day.

On the evening of admission day 30, repeat catheter venography revealed rethrombosis of the left TS and persistent large volume clot within the SSS. In addition, stenosis near the junction of the left TS and sigmoid sinus was noted (Figure 2B). The decision was made to perform suction thrombectomy utilizing a reperfusion catheter (ACE68^TM^ Reperfusion Catheter, Penumbra, USA) with a suction pump (Pump MAX^TM^, Penumbra, USA). Suction thrombectomy of the SSS, left TS, and sigmoid sinus yielded a large volume of clot. The in situ stenosis persisted at the junction of the left transverse and sigmoid sinuses. Thus, angioplasty was performed utilizing a 5 mm x 20 mm balloon (Viatrac^TM^ 14 Plus, Abbott, USA) followed by successful deployment of an 8 mm x 40 mm self-expanding stent (Zilver®, Cook Medical, USA) with good venographic outcome (Figure 2C).

The patient experienced improvement in visual deficits and headache the next day, with resolution of these symptoms by day 32. She was converted from a heparin drip to a twice daily dose of enoxaparin 1 mg/kg and discharged home on day 36 with only mild blurry vision.

**Figure 3:**
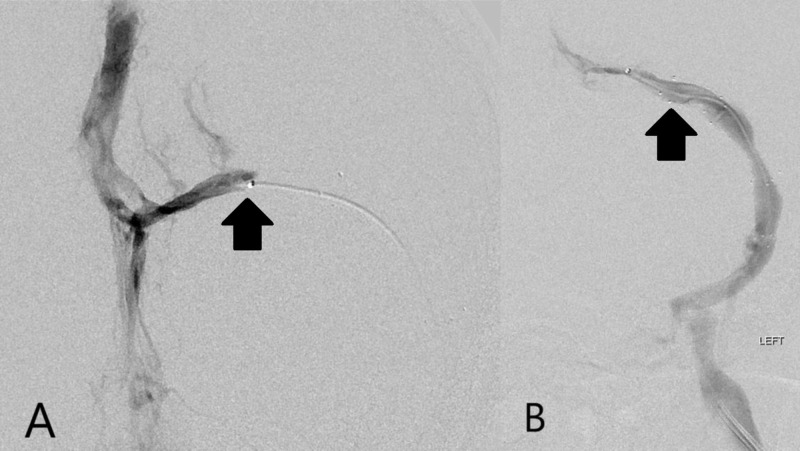
Recurrent transverse sinus occlusion with second treatment with angioplasty and stent placement. A) Digital subtraction angiography image from left transverse sinus venography after recurrence of symptoms demonstrating complete re-occlusion (arrow) of the left transverse sinus and indwelling stent (left transverse sinus pressure 53 mmHg). B) Digital subtraction angiography image after angioplasty and placement of a second self-expanding stent (arrow) demonstrating recanalization of the left transverse sinus with restoration of outflow (left transverse sinus pressure 22 mmHg).

Eight days after discharge, the patient was readmitted with a lower extremity hematoma, likely a complication of systemic anticoagulation. Upon admission, she reported worsened blurry vision and intermittent headaches. These prompted reevaluation of the dural venous sinuses by CT venography. This revealed in-stent thrombosis of the left TS with ~75% stenosis and nonocclusive thrombus within the SSS along with the confluence of sinuses. She was then discharged home again, returning shortly thereafter as an outpatient for catheter venography. This demonstrated the progression of thrombosis to completely occlusive near the junction of the left TS and sigmoid sinus, along with markedly elevated pressure within the left TS of 53 mmHg (Figure 3A).

Thus, suction thrombectomy proved of limited benefit. Therefore, balloon angioplasty was performed and a second self-expanding stent was placed. This procedure demonstrated excellent venographic outcome and significant decrease in measured pressure within the left TS to 22 mmHg (Figure 3B). The headaches quickly resolved, although mild blurry vision persisted.

One month following the placement of the second stent, follow-up imaging confirmed patency of the dural sinuses and left TS stents. Twelve weeks after placement of the second stent, the patient remains headache-free with only mild blurry vision.

## Discussion

Many risk factors for DST have been previously described, including pregnancy, low CSF volume, infection, trauma, hereditary thrombophilia, hyperhomocysteinemia, elevated estrogen levels, and other hematological factors [1-4]. Approximately 7.4% of DST occurs in patients with a known malignancy. Malignancy-related DST may be due to direct venous compression by tumor, venous invasion, or hypercoagulability [3-4]. Chang et al. reported DST in association with AML in a 40-year-old woman who had AML-associated hyperhomocysteinemia [5]. The DST was successfully managed with systemic anticoagulation, while endovascular intervention was not deemed necessary [5]. Treatment of DST is usually focused on anticoagulation to treat the existing thrombus and prevent new thrombosis [1, 6-8]. Endovascular intervention is reserved for severe or anticoagulation-refractory cases [6-7, 9]. In our presented case, the massive clot burden and severe progressive symptoms prompted the decision to pursue aggressive endovascular management. The patient’s AML presented a challenge to safe and effective management. Underlying malignancy is likely to limit effectiveness of medical therapy alone, while also increasing the risk of post-procedural complications [2]. Despite these challenges, intervention was deemed necessary in this case as a vision-saving and potentially life-saving measure. Further investigation is needed to fully examine the role of endovascular intervention in complex clinical scenarios involving DST. However, our case provides an example of the successful role for aggressive intervention in a complex and challenging combination of DST-associated disease processes.

## Conclusions

Individuals with malignancies are at an increased risk for developing DST. Although systemic anticoagulation is considered first-line therapy, endovascular intervention may be necessary if DST is refractory to medical therapy, the patient’s clinical course deteriorates, or if the presenting symptoms are particularly severe. A favorable outcome was achieved in this case through aggressive endovascular intervention despite the massive clot burden and additional challenges presented by the underlying malignancy.
